# A new murine ileostomy model: recycling stool prevents intestinal atrophy in the distal side of ileostomy

**DOI:** 10.20407/fmj.2020-003

**Published:** 2020-10-10

**Authors:** Naoko Uga, Masashi Nakatani, Aya Yoshimura, Kanako Kumamoto, Kunihiro Tsuchida, Shizuko Nagao, Tomonori Tsuchiya, Yasuhiro Kondo, Atsuki Naoe, Shunsuke Watanabe, Toshihiro Yasui, Fujio Hara, Tatsuya Suzuki

**Affiliations:** 1 Department of Pediatric Surgery, Fujita Health University, School of Medicine, Toyoake, Aichi, Japan; 2 Division for Therapies against Intractable Diseases, Institute for Comprehensive Medical Science, Fujita Health University, Toyoake, Aichi, Japan; 3 Education and Research Center of Animal Models for Human Diseases, Fujita Health University, Toyoake, Aichi, Japan

**Keywords:** Stoma, Recycling stool, Intestinal atrophy, Ileostomy, Cell proliferation

## Abstract

**Objectives:**

Proximal stoma creation in neonates results in growth failure and distal intestinal atrophy. “Recycling stool” consists of stool injection from the proximal limb to the distal limb of a stoma. Because this method may prevent distal bowel atrophy and increase body weight, we investigated the effects of recycling stool upon distal intestinal mucosa by generating an ileostomy model in rats.

**Methods:**

An ileostomy was created 5 cm proximal to the cecum in male Wistar/ST rats. Discharged stool or saline was injected into the distal limb, twice per day for 7 days. The intestinal adaptation was assessed by measuring the villus height and counting goblet cell number. Proliferation and apoptosis were analyzed by Ki67 and TUNEL immunostaining.

**Results:**

The ratios of the height of the distal villi (D) to the that of proximal villi (P) were 0.97 (median [range] of D and P length: 421 [240–729] μm and 436 [294–638] μm, *P*<0.05) in the stool-injected group and 0.81 in the saline-injected group (442 [315–641] μm and 548 [236–776] μm, *P*<0.05). Compared with the saline-injected group, the stool-injected group showed elevated numbers of goblet cells (3.6 [2.0–7.6] vs. 4.9 [2.4–7.5] cells/100-μm villus length) and Ki67-positive cells (26.8% [13.8%–35.4%] vs. 40.1% [31.2%–45.7%]), along with a reduced number of apoptotic cells (5.0 [2.0–14.0] vs. 4.0 [1.0–9.0] cells/100-μm villus length).

**Conclusions:**

Recycling stool prevented distal intestinal atrophy; this experimental design may facilitate further studies concerning alternative methods to prevent intestinal atrophy and growth failure.

## Introduction

Various neonatal disorders, such as necrotizing enterocolitis, local intestinal perforation, meconium ileus, require the creation of a small intestinal stoma.^1,2^ A proximal stoma secretes large quantities of intestinal fluids containing unabsorbed nutrients and water, which results in dehydration, excessive sodium loss, and electrolyte imbalance.^1,3^ Patients in this situation also suffer from malnutrition, which leads to poor growth, and need continuous total parenteral nutrition (TPN). However, TPN often can cause cholestasis and liver failure. Additionally, thrombosis and repeated infection may occur at the TPN catheter set. Thus, the patients cannot be discharged promptly and experience poor quality of life, with a high risk of mortality.

Deprivation of luminal nutrients leads to adaptive changes in response to a new environment. One of the main manifestation of these changes is reduction of intestinal villus height. Mucus in the distal part of a stoma also exhibits atrophy.^4^ The decreased bowel circumference is a result of intestinal atrophy, which leads to difficulty in subsequent anastomosis procedures.

“Recycling stool” refers to the injection of stool from the proximal limb of a stoma to its distal limb1. In the 1980s, Puppala et al. first described how continuous stool transport in the distal intestine of a stoma by using a pump and the method led to gain in body weight (BW) in two pediatric patients.^5^ In the 1990s, Schäfer et al. performed stool recycling as treatment for very-low-birth-weight infants.^6^ Currently, intermittent stool recycling is often performed and/or surgical care of pediatric patients. Several investigations have reported that in neonates with high enterostomy, recycling stool facilitated BW gain, reducing TPN requirements and TPN-associated cholestasis. Additionally, a smaller discrepancy in bowel-end size was observed due to intermittent transport of stool to the distal fistula of the stoma.^2,3,7,8^ Stool passage through the distal intestine is believed to stimulate the absorptive surface of the mucous membrane and contribute to mucosal growth, thereby, preventing distal bowel atrophy.

Previous investigations in rats have concluded that fasting led to intestinal mucous atrophy and that refeeding reversed the atrophy, according to histological and molecular analyses.^9,10^ However, little is known regarding the histological response that occurs after creation of an enterostomy and subsequent recycling stool. Especially, neonates subjected to recycling stool are affected by various factors, such as gestational age, birth weight, and disease. Therefore, it is difficult to analyze formally and statistically as these procedures are only be performed in a few patients. This is probably why a regimen of stool recycling has not yet been established. Thus, we investigated the effect of recycling stool using a rat model. To the best of our knowledge, this is the first report concerning the effects of recycling bowel content on intestinal mucosa using an ileostomy animal model. This model may be useful in establishment of a protocol for recycling stool.

## Methods

### Animals

Male Wistar/ST rats (8 weeks of age, 200–250 g BW) were purchased from Japan SLC (Shizuoka, Japan). Rats were housed in individual plastic cages under a 12-h light-dark cycle at a room temperature of 21±2°C. Rats were provided water and standard chow (MF; Oriental Yeast Co. Ltd., Kyoto, Japan) ad libitum.

### Statement regarding animal welfare

All applicable international, national, and institutional guidelines for the care and use of animals were followed. All procedures were performed in accordance with the ethical standards of Fujita Health University, where the study was conducted (Animal Care and Use Committee of Fujita Health University, approval number: AP17007-MD2).

### Surgical procedure and stool recycling from ileostomy

After 2–5 days of acclimatization, rats were divided into two groups: ileostomy and sham. Surgical procedures were performed under anesthesia with a mixture of medetomidine (0.375 mg/kg BW), midazolam (2.0 mg/kg BW), and butorphanol (2.5 mg/kg BW). To lower the risk of infections, cefotaxime (20 mg/kg BW) was administered before surgery.

A loop ileostomy was constructed on the abdominal wall using a modified version of the method reported by Volenec et al.^11^ The abdomen was shaved and disinfected with 70% ethanol. A 2 cm midline incision was made on the upper abdomen. The ileum was pulled ventrally, 5 cm proximal to the cecum. The right and left abdominal walls were sutured by 5-0 nylon, under the (now external) ileum loop, to anchor the loop. Two-thirds of the circumference of the ileum was transected at the ventral side, and a 2 mm incision was made to the opposite sides of mesenteries at both ends of the limb to promote stool injection. Each edge of the ileum limb was sutured through the abdominal wall in four places with 5-0 nylon. Finally, the rostral and caudal edges and each side of the bridge were fixed to the skin with 7-0 nylon to prevent rupture. A sham operation with intact ileum was also performed. A 2 cm midline incision was made at the upper abdomen, then closed with a double-deck running suture using 5-0 nylon.

### Experimental design

The following two experiments were carried out in the present study.

### Experiment 1

Rats were divided into two group: ileostomy and sham operation. Rats had access to water and chow ad libitum. Tissue samples were collected 7 days after surgery.

### Experiment 2

Rats were divided into three groups: stool, saline, and sham. In the stool group, 24 h after the surgical procedure, discharged stool from the proximal limb was injected into the distal limb of the ileostomy, twice per day (08:00 and 20:00) for 7 days. The collected stool was approximately 8 g per in daytime. Therefore, stool was suspended in saline; 4 mL of this suspension was injected using a syringe into the distal limb of the ileostomy of the rat from which the stool was initially collected. In the saline group, 4 mL of warmed saline was injected into the distal limb of the ileostomy instead of stool. In the sham group, rats underwent a sham surgical procedure. In this experiment, an original stoma pouch was worn firmly to each rat to collect stool between 08:00 and 20:00 in all groups.

### Collection of intestinal tissue

After perfusion fixation with 4% paraformaldehyde, the entire small intestine and colon were removed carefully. The lumen of the intestine was flushed with ice-cold phosphate-buffered saline without Ca2+ and Mg2+ (PBS (–)) to clear feces from the intestinal lumen. For histology, sections of intestinal tissue were cut out at 0.5–3.5 cm from the proximal and distal sides of the ileostomy. The two sides of each section were sliced and used of cell morphology. In the sham group, two tissue samples were taken at positions identical to those used in the ileostomy group (0.5–3.5 cm from the incision position for both proximal and distal ends of the ileostomy). Tissue samples were pinned onto a corkboard to prevent contraction and distortion. they were immersed in 4% paraformaldehyde overnight and embedded in paraffin. All segments were cut transversely at a thickness of 4 μm and processed by standard hematoxylin and eosin (H&E) staining for morphology and immunohistochemistry analyses.

### Morphology of intestinal tissue

Images of H&E-stained sections were taken using a BX43 light microscope (Olympus, Tokyo, Japan) with a DP70 digital camera (Olympus) and DP controller software (Olympus) at ×100 magnification. The height was measured from the tip of the villus to the muscularis mucosae using ImageJ (National Institutes of Health, Bethesda, MD, USA). Using each side of the section, villus height was measured for 20 well-oriented villi. Therefore, an average of 40 villi was measured in each section. The effect of ileostomy creation upon the number of goblet cells was determined by measurement of the mean number of goblet cells per 100 μm villus column in well-oriented villi in each tissue section.

### Immunohistochemistry analysis

Immunostaining of Ki67 was used to assess cell proliferation in villi. After deparaffinization of paraffin sections, they were heated in citrate buffer solution (pH 6.0) to unmask antigens. After cooling, each slide was washed with PBS (–). Sections were incubated with 4% Block ACE (DS Pharma Biomedical, Osaka, Japan) in PBS (–) containing 0.05% Tween 20 (PBS-T) for 2 h at room temperature, then washed three times with PBS-T. A primary antibody against Ki67 (GTX16667; 1:100 dilution; GeneTex, Los Angeles, CA, USA) was used. Sections were again washed three times with PBS-T. After secondary antibody (Alexa Fluor 555 anti-rabbit IgG; 1:500 dilution; Thermo Fisher Scientific, Waltham, MA, USA) was added, the slides were incubated.

To detect apoptotic cells in intestinal epithelial villi, slides were stained by using the terminal deoxynucleotidyl transferase dUTP nick-end labeling (TUNEL). Staining was performed using the MEBSTAIN^®^ Apoptosis TUNEL Kit III, in accordance with the manufacturer’s instructions (MBL, Aichi, Japan). In brief, sections were deparaffinized, then incubated with proteinase K. Subsequently, terminal deoxynucleotidyl transferase (TdT) Buffer II was applied to sections. TdT Buffer II was then removed, and TdT solution was added and incubated. Slides were rinsed in TB Solution, then washed with distilled water. Blocking solution was added to the slides and incubated. Specimens were reacted with avidin-(4,6-dichlorotriazinyl) aminofluorescein solution and washed with distilled water.

ProLong™ Diamond Antifade Mountant with 4',6-diamidino-2-phenylindole (DAPI) (Thermo Fisher Scientific) was used for mounting. Images were obtained using a BX51 fluorescence microscope (Olympus) with a DP71 digital camera (Olympus) and DP controller software at ×200 magnification. Ki67-positive cells were counted from three fields of the distal ileum for each rat. TUNEL-positive cells were counted by examining 100 μm villus tip in well-oriented villi in each distal tissue section.

### Statistical analysis

Data are shown as the median (range). In experiment 1, the Mann–Whitney U test was used to determine mean differences between the ileostomy and sham groups. In experiment 2, the Kruskal–Wallis test with post hoc Scheffe tests was used to determine differences among the three groups. Differences with *P*<0.05 were considered statistically significant.

## Results

### Changes in BW

**Figure 1:**
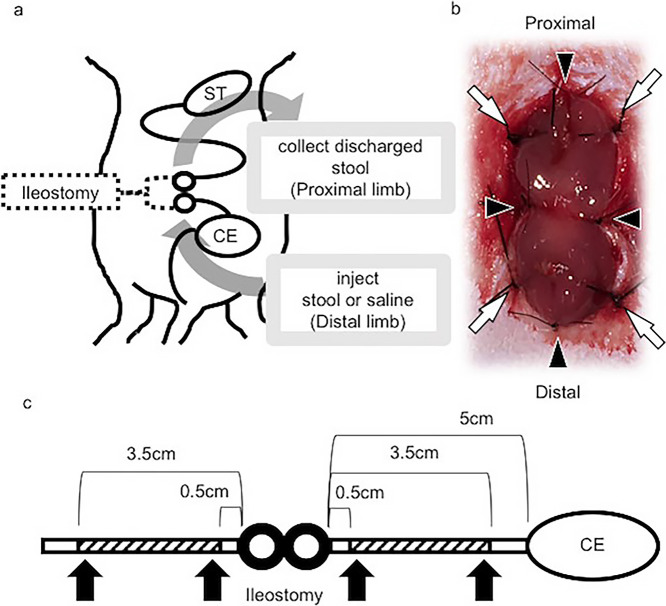
Schematic of ileostomy in rats a) Ileostomy was constructed 5 cm from cecum. Stool to be recycled was discharged from proximal limb and injected into distal limb of ileostomy. ST: stomach. CE: cecum. b) Image of ileostomy. Borders of ileum limb were sutured with 5-0 nylon through abdominal wall at four locations (arrows). Rostral and caudal rims and each side of bridge between each 5-0 nylon suture were fixed to skin with 7-0 nylon (arrowheads). c) Intestinal sections for histology were cut at 0.5–3.5 cm from ileostomy, at both proximal and distal sides (shaded portion). Both sides of each section were sliced and used for cell morphology analysis (arrows).

Ileostomy images are shown in Figure 1a and b. Table 1 demonstrates changes in BW before and after surgery. In experiment 1, the sham group showed BW gain of 6.7%; the ileostomy group showed gradual reduction in BW, reaching an overall loss of 34.7%. In experiment 2, the saline and stool groups both lost BW. There was no significant difference in BW loss between the saline group (35.1% loss) and stool group (39.5% loss). The sham group showed elevated BW (10.9% gain), as observed in experiment 1.

### Histologic changes in the small intestine

### Villus height

**Figure 2:**
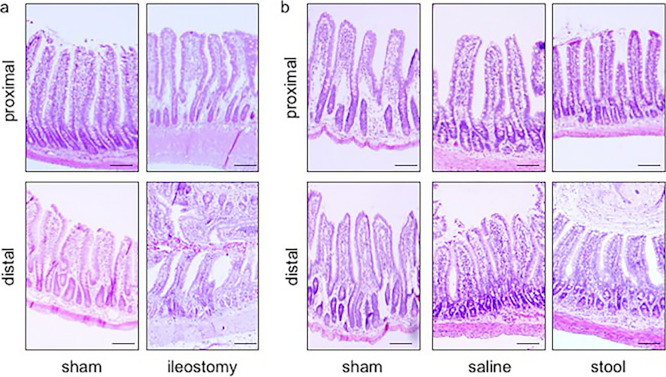
Intestinal morphology in ileostomy-operated rats Sections of proximal and distal ileum were prepared and stained with H&E (scale bar=100 mm). a) Sham and ileostomy groups in experiment 1. b) Sham, saline, and stool groups in experiment 2.

We undertook H&E staining of the proximal and distal limbs of the ileostomy (Figure 1c and Figure 2). In general, the height of an intestinal villus decreases gradually from the jejunum to the ileum end.^12^ So, changes in villus height were expressed as the ratio of the height of distal villi (D) to the that of proximal villi (P); thus, the ratio was denoted as “D/P” (Table 2). The D/P ratio showed only a slight difference in sham groups, compared between experiments 1 and 2. There was a considerable difference in the D/P ratio of ileostomy-operated rats without stool injection (experiment 1: 0.91 in the sham group vs. 0.86 in the ileostomy group; experiment 2: 1.02 in the sham group vs. 0.81 in the ileostomy group with saline injection). The difference in D/P ratio was not large when collected stool were injected into the ileum through the stoma (1.02 in the sham group *vs.* 0.97 in the ileostomy group with stool injection). This observation suggested that stool injection inhibited progressive atrophy in the distal intestinal mucosa.

### Goblet cells

Goblet cells are flask-shaped cells that secrete mucin to protect the luminal epithelial surface from infection by pathogenic bacteria. Mucin can also ease the passage of feces.^13,14^ In this study, we counted numbers of goblet cells in the distal limb of the ileostomy.

**Figure 3:**
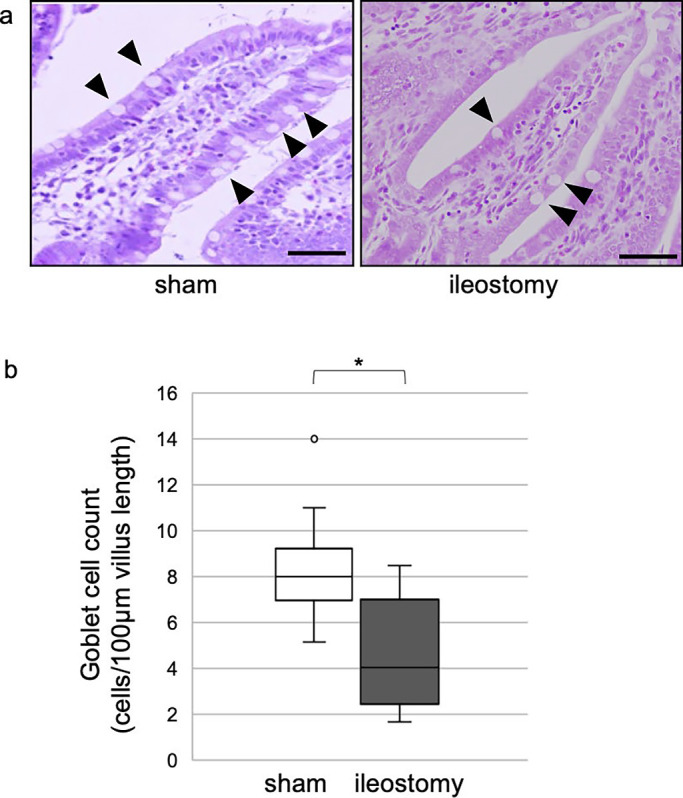
Numbers of goblet cells in experiment 1 a) H&E stained distal ileum from sham and ileostomy groups in experiment 1 (arrowheads=goblet cells) (scale bar=50 mm). b) Quantitative analysis with Mann–Whitney U test (median from four villi of each rat, n=4). *P<0.05.

**Figure 4:**
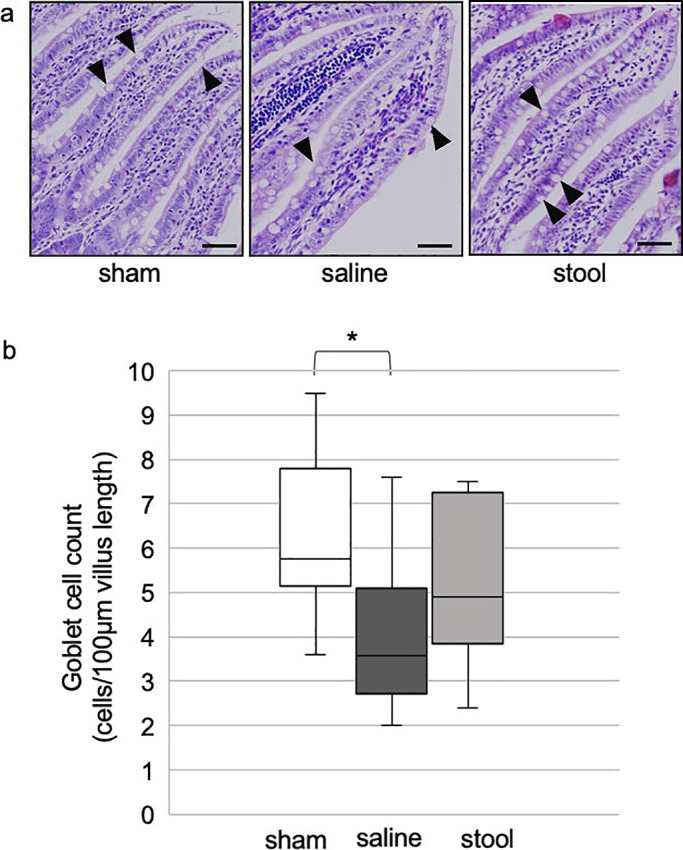
Numbers of goblet cells in experiment 2 a) H&E stained distal ileum from sham, saline, and stool groups in experiment 2 (arrowheads=goblet cells) (scale bar=50 mm). b) Quantitative analysis with Kruskal–Wallis test (median from four villi of each rat, n=3). *P<0.05

In experiment 1, the number of goblet cells significantly decreased in the ileostomy group (8.0 [5.1–14.0] in the sham group *vs.* 4.1 [1.7–8.5] in the ileostomy group, *P*<0.05) (Figure 3). In experiment 2, although saline-treated rats showed a reduction in the number of goblet cells, rats injected with stool showed no significant difference in the number of goblet cells (5.8 [3.6–9.5] in the sham group, 3.6 [2.0–7.6] in the saline group, and 4.9 [2.4–7.5] in the stool group; *P*<0.05 for sham *vs.* saline group) (Figure 4).

### Proliferation and apoptosis

**Figure 5:**
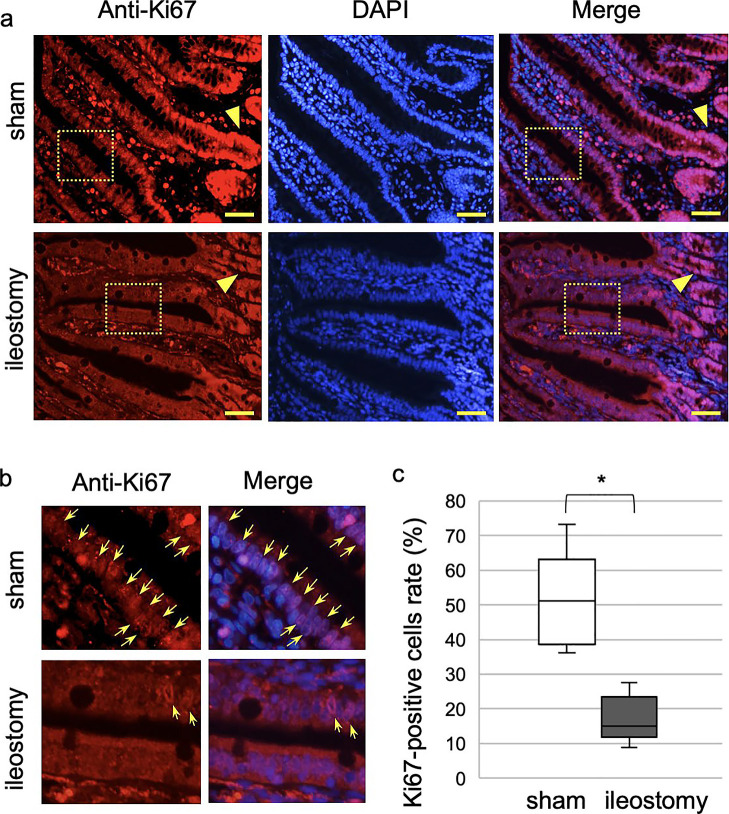
Ki67 immunostaining in experiment 1 a) Low and b) high magnifications of Ki67 (red)-stained ileum sections in sham and ileostomy groups (scale bar=50 mm). Nuclei were stained with DAPI (blue). Arrowheads indicate crypts (arrows=Ki67-positive nuclei). c) Quantitative analysis with Mann–Whitney U test (median of three fields of each rat, n=4). *P<0.05.

**Figure 6:**
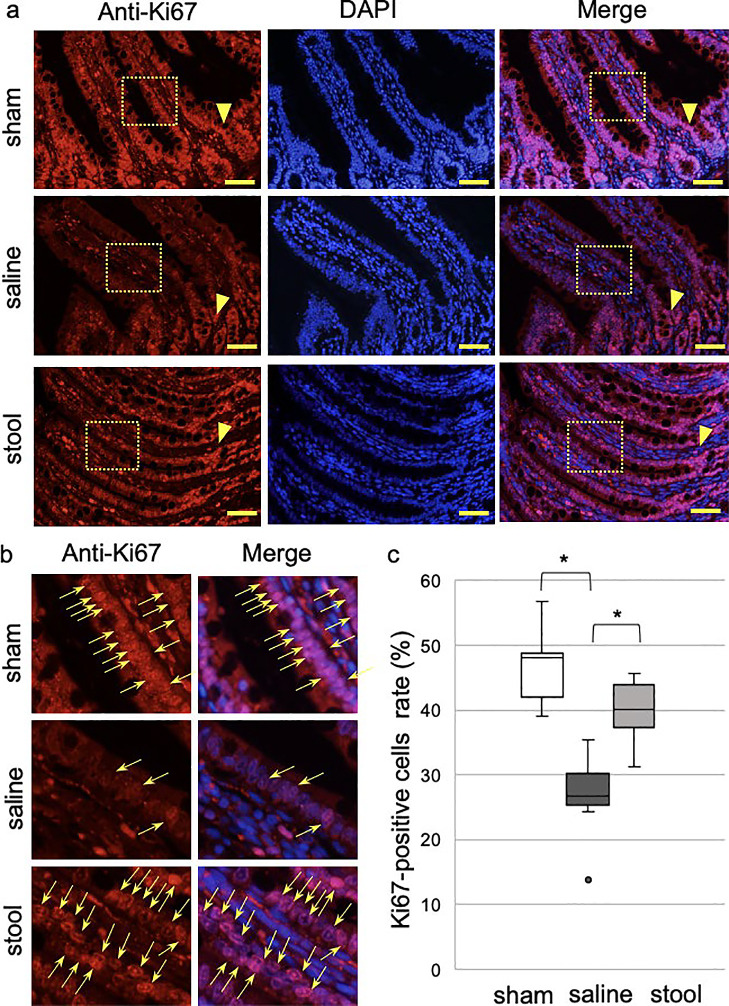
Ki67 immunostaining in experiment 2 a) Low and b) high magnifications of Ki67 (red)-stained ileum sections in sham, saline, and stool groups (scale bar=50 mm). Nuclei were stained with DAPI (blue). Arrowheads indicate crypts (arrows=Ki67-positive nuclei). c) Quantitative analysis with Kruskal-Wallis test (median from three fields of each rat, n=3). *P<0.05.

To evaluate the effect of stool in the intestinal lumen on cell proliferation, Ki67 staining of distal mucosa was performed. In experiment 1, Ki67-positive cells in the sham group were observed in the crypts (Figure 5a, arrowheads), as well as in the the bottom and middle of villi (Figure 5b). In contrast, nearly all Ki67-positive cells in the ileostomy group were located around the crypts. Stoma creation significantly reduced the numbers of Ki67-positive cells in the distal intestine (51.1% [36.2%–73.2%] in the sham group *vs.* 15.0% [8.8%–27.6%] in the ileostomy group, *P*<0.05) (Figure 5c). In experiment 2, Ki67-positive cells were observed in the lower halves of the villi in the stool group (Figure 6a and b) as well as in the sham group. In contrast, in the saline-treated group, Ki67-positive cells were located mainly around the crypt. The percentage of Ki67-positive cells in the distal intestine was significantly lower in the saline group (26.8% [13.8%–35.4%]) than in the sham group (48.0% [39.0%–56.7%], *P*<0.05) or stool group (40.1% [31.2%–45.7%], *P*<0.05). The percentage of the stool group was no significantly change between that of the sham group (Figure 6c).

**Figure 7:**
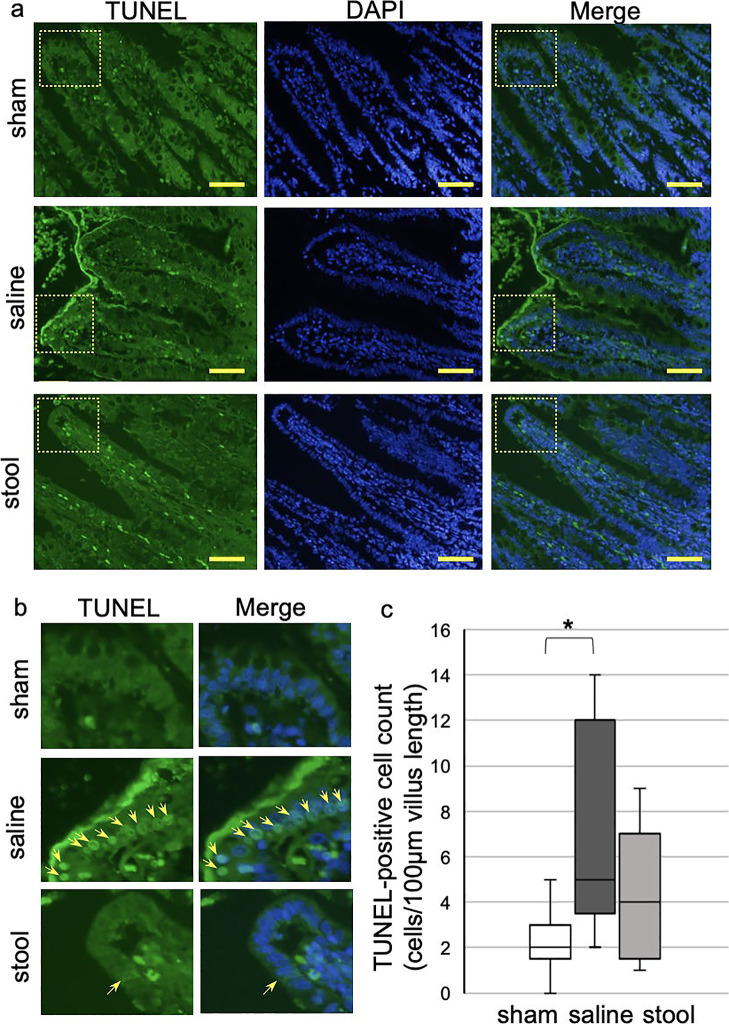
TUNEL immunostaining in experiment 2 a) Low and b) high magnifications of TUNEL (green)-stained distal ileum sections in sham, saline, and stool groups (scale bar=50 mm). Nuclei were stained with DAPI (blue). Arrows indicate TUNEL-positive cells. c) Quantitative analysis with Kruskal-Wallis test (median from three villus tip of each rat, n=3). *P<0.05.

TUNEL staining is employed to detect apoptotic cells. Apoptotic cells were observed mainly on villus tips (Figure 7a and b). These observations suggested that apoptotic cells had exfoliated to the lumen. In experiment 2, the number of TUNEL-positive cells in each 100 µm villus tip was lower in the stool group than in the saline group (2.0 [0–5.0] cells/100 μm villus tip in the sham group, 5.0 [2.0–14.0] cells/100 μm villus tip in the saline group, and 4.0 [1.0–9.0] cells/100-μm villus tip in the stool group; *P*<0.05 in sham *vs.* saline group) (Figure 7c). Little difference of TUNEL-positive cells in the lamina propria was observed in the three groups.

## Discussion

Recycling stool from the proximal limb to the distal limb is often performed in neonates in whom high duodenostomy or jejunostomy has been created. This strategy is believed to prevent atrophy by stimulating unused mucosa in the distal limb of an enterostomy. Additionally, it reduces the requirement for TPN and allows BW gain.^1,3,5,8^ To our knowledge, little is known about the histological effect of recycling stool on the intestinal mucosa of the distal limb.

Reportedly, fasting reduces intestinal weight and results in diminished mucosal cell proliferation in rats.^10,15^ Dunel-Erb et al. observed that, after fasting in rats, villus height significantly decreased, and that smooth muscle cells shrank in the lamina propria, which is connective tissue.^9^ These morphologic changes in villi during fasting increased the efficiency of absorption by enlarging the villus surface relative to the volume. The atrophy must be the result of deprivation of nutrition in the lumen and/or an indirect effect of decreased pancreaticobiliary secretions, which are considered trophic to intestinal mucosa^4^. As observed during fasting, atrophy can occur at the distal intestinal mucosa of a stoma because digested foods are absent at the distal limb. In rats, enteral nutrition produces enterotropic effects that restore mucosal atrophy and maintain mucosal integrity.^10^ Refeeding after fasting has been contributed to the recovery of villus height.^9^

Contrary to our expectations, rats in the stool group in experiment 2 did not gain BW. We considered that the amount of stool was insufficient, because the amount of collected stool in this experiment was not all of the stool discharged during the day. However, we observed a histological effect of stool on the intestinal atrophy induced by stoma creation. The significant decrease in villus height in the distal mucosa of rats in the saline group was not shown among rats in the stool group. One of the most important energy source for enterocytes in the small intestine is glutamine, which nourish the intestine from the circulation through the basolateral membrane, and from the lumen through the brush border of the epithelial cells.^16,17^ In this study rats did not receive TPN. Hence, nutrition contained in the stool such as glutamine may have increased villus height. In addition, several enteral nutrients or breast milk, which contents glutamine, could increase villus height.

Homeostasis of the intestinal mucosa depends on a balance between the cell proliferation and apoptosis.^15^ The turnover of enterocytes is regulated by immature cells in intestinal crypts, which differentiate and move to the tip of the villus in 3–6 days.^15,18^ If the intestinal mucosa undergoes continuous cell turnover, this proliferation must be counterbalanced by a similar rate of apoptosis.^18,19^ In experiment 1, we showed that stoma creation decreased the number of Ki67-positive cells. This observation suggested that the absence of intestinal luminal content resulted in inhibition of cell proliferation. In experiment 2, there were more Ki67-positive cells in the stool group than in the saline group. However, the sham and stool groups exhibited Ki67-positive cells in the crypts and intermediate sections of villi. Several studies have shown that cell proliferation occurs within the limited bottom part of a crypt, and that cells migrate upward to the villus tip.^9,13^ Gomes et al. reported that fasting caused a delay in cell migration in the villus.^20^ Our results indicated that stool recycling suppressed a delay in migration, and stimulated cell proliferation which led to increase villus height.

Apoptosis is known to occur mainly at the tips of villi^13^ and apoptotic cells are exfoliated into the lumen of the intestine,^19^ a hypothesis that is consistent with a luminal route of cell turnover. Iwakiri et al. and Boza et al. hypothesized that during fasting, when the growth and proliferation of cells are at the lowest, the apoptosis of intestinal cells is triggered.^10,21^ In rats, intestinal cell apoptosis increases in the presence of glutamine deprivation.^22^ In experiment 2, apoptotic cells in the saline group were observed at the tips of villi. However, the stool group showed few TUNEL-positive cells, even at the tips of villi. These results suggest that recycling stool to the intestinal lumen reduced epithelial cell apoptosis.

We observed reductions in the numbers of goblet cells in the ileostomy group in experiment 1 and saline group in experiment 2. Thus, stool injection through the stoma may have suppressed the reduction in goblet cell number induced by ileostomy creation. It has been reported that goblet cells differentiate from the stem cells in the crypt.^13^ Hence, we suspect that the reductions in the number of goblet cells resulted from diminished cell proliferation or migration to villi. Goblet cells produce and secrete mucins, especially MUC2.^14,23^ Additionally, goblet cells play a role in the immune system: they function as “gatekeepers” that deter oral antigens. Therefore, an increase number of goblet cells helps to inhibit infection by pathogens present in luminal contents.

However, studies have described the differentiation of absorption efficiency in various segments of the small intestine of rats.^24,25^ Such disparities may contribute to the distinct outcomes observed after duodenostomy or jejunostomy.

## Conclusions

We investigated the short-term histological effect of stool recycling, from the proximal limb to the distal limb of an ileostomy model in rats. Stool passage through the distal intestine prevented mucosal atrophy. Further studies are needed regarding the frequency and amount of stool injections needed to establish a more efficient regimen for recycling stool. If other substances, such as enteral nutrients, can be used instead of stool, and these are found to have a similar or greater effectiveness in preventing intestinal mucosa atrophy, this could be more beneficial for patients. In the future, alternative substances than stool from the patient may be sought by using this animal model.

## Figures and Tables

**Table1:** Body weight (g) changes in sham and ileostomy-operated rats

Experiment 1	Day of surgery median (range)	7 days after surgery median (range)
Sham group (n=4)	284 (147–193)	303 (278–315)
Ileostomy group (n=4)	268 (240–293)	175 (158–192)*
Experiment 2	Day of surgery median (range)	7 days after surgery median (range)
Sham group (n=3)	248 (234–157)	275 (294–272)
Saline group (n=3)	262 (242–265)	170 (165–182)*
Stool group (n=3)	261 (251–280)	158 (155–187)*

The number of animals is given in parentheses. *; *P*<0.05 vs sham group, with the Mann–Whitney U test.

**Table2:** Ileal villus height (μm) at 7 days after surgery

Experiment 1	Proximal median (range)	Distal median (range)	Index (D/P)
Sham group (n=4)	422 (246–775)	383 (282–644)*	**0.91**
Ileostomy group (n=4)	425 (222–625)	364 (225–536)*	**0.86**
Experiment 2	Proximal median (range)	Distal median (range)	Index (D/P)
Sham group (n=3)	390 (210–578)	397 (215–718)	**1.02**
Saline group (n=3)	548 (236–776)	442 (315–641)*	**0.81**
Stool group (n=3)	436 (294–638)	421 (240–729)	**0.97**

Number of animals is shown in parentheses. Median (range) from 160 villi and 120 villi in each section in Experiments 1 and 2, respectively. Ratio of height of distal villi (D) to height of proximal villi (P) *; *P*<0.05 vs proximal, compared via Mann–Whitney U test in Experiment 1, and Kruskal–Wallis test with post hoc Scheffe tests in Experiment 2.
